# Clinical Application of Human Induced Pluripotent Stem Cell-Derived Organoids as an Alternative to Organ Transplantation

**DOI:** 10.1155/2021/6632160

**Published:** 2021-02-24

**Authors:** Gabriella Shih Ping Hsia, Joyce Esposito, Letícia Alves da Rocha, Sofia Lígia Guimarães Ramos, Oswaldo Keith Okamoto

**Affiliations:** ^1^Human Genome and Stem Cell Research Center, Departamento de Genética e Biologia Evolutiva, Instituto de Biociências, Universidade de São Paulo, SP, Brazil; ^2^Genetics Unit, Instituto da Criança, Hospital das Clínicas, Faculdade de Medicina, Universidade de São Paulo, SP, Brazil; ^3^Hemotherapy and Cell Therapy Department, Hospital Israelita Albert Einstein, São Paulo, SP, Brazil

## Abstract

Transplantation is essential and crucial for individuals suffering from end-stage organ failure diseases. However, there are still many challenges regarding these procedures, such as high rates of organ rejection, shortage of organ donors, and long waiting lines. Thus, investments and efforts to develop laboratory-grown organs have increased over the past years, and with the recent progress in regenerative medicine, growing organs *in vitro* might be a reality within the next decades. One of the many different strategies to address this issue relies on organoid technology, a miniaturized and simplified version of an organ. Here, we address recent progress on organoid research, focusing on transplantation of intestine, retina, kidney, liver, pancreas, brain, lung, and heart organoids. Also, we discuss the main outcomes after organoid transplantation, common challenges faced by these promising regenerative medicine approaches, and future perspectives on the field.

## 1. Introduction

Organ transplantation is still an important and necessary procedure that increases overall survival of many patients with organ failure diseases. It has been largely reported that organ transplantation improves the quality of life (QoL) of these patients. For instance, kidney transplantation provides more benefits and a better QoL for patients compared to hemodialysis [1–7]. Even though medicine and technology have advanced greatly over the past years, organ transplantation still faces many issues: ethical and religious concerns (since many organs are derived from brain-dead or non-heart-beating donors); organ trafficking; elevated risk of organ rejection, the possibility of health complications for living donors and receptors posttransplantation; the necessity of additional tests before transplantation; continuous use of immunosuppressive drugs/medications; and psychological impacts [8, 9]. Even when most conditions are favorable for transplantation, the number of available donors usually does not cover the number of patients in need of a donation. For instance, in the United States, a survey conducted in 2013 revealed that more than 116,000 patients were on the waiting list for transplantation, but only 28,000 underwent the procedure [10–15].

Some of these issues are the reason for decreased patients' QoL posttransplantation [16–19]. Thus, there has been an urgent need for new strategies for tissue repair and organ replacement. Over the past years, the development of laboratory-grown organs has been the focus of many types of research.

In 2006, a big step towards this goal was made by Yamanaka and collaborators [20] with the advent of induced pluripotent stem cells (iPSCs), opening many new possibilities for the emergence of other technologies, such as 3D bioprinting and organoid development, making the production of organ-like structures in the laboratory a close reality. Here, we discuss how these novel technologies have evolved towards organoid development, new insights in the transplantation of different types of organoids, its outcomes, and challenges.

## 2. Manipulating Cell Identity: The Foundation

Cell manipulation is an essential tool to provide efficient and reliable biological information, allowing the study of various human diseases through a system that mimics *in vivo* physiological conditions [21, 22]. The first attempt of cell manipulation dates back to 1907, when Ross Harrison not only developed an innovative *in vitro* method, isolating frog embryo nerve fibers, but also maintained them successfully in culture [23]. Later, in 1955, King and Briggs developed a method to transfer the nuclei of embryonic cells into enucleated frog eggs [24]. In 1962, Gurdon demonstrated that cell specialization is a reversible process; the immature cell nucleus of a frog egg cell was replaced by a mature intestinal cell nucleus, generating a zygote-like cell that successfully developed into a normal tadpole [25]. In 1981, Evans and Kaufman obtained embryonic stem cells (ESCs) from mouse embryos [26], and in 1995, Thomson et al. isolated the first ESCs from primates [27].

These achievements contributed to the development of methods to derive and cultivate ESCs from human embryos, which started in 1998 [28] and continues until nowadays, leading to major breakthroughs, such as the discovery of Yamanaka and colleagues in 2006 on how to reprogram somatic cells into pluripotent stem cells (PSCs) [20]. The authors discovered that the ectopic expression of four defined factors, *Oct3-4*, *c-Myc*, *Sox2*, and *Klf4*, was necessary and sufficient to reprogram human adult cells into a pluripotent state, producing iPSCs [20]. This revolutionary technology opened a myriad of possible applications impacting personalized medicine, drug screening, and human disease modeling, without ethical hurdles imposed by therapeutic cloning and the use of human embryos. Furthermore, due to the possibility of generating patient-specific cells from iPSCs, this discovery also brought a possible solution to circumvent immune rejection, one of the main complications in transplantation.

## 3. Organoids: Why Use a Tridimensional System?

Many clinically oriented cell therapy studies have reported controversial results about therapeutic evidence and adverse events [29, 30]. Most early studies rely on two-dimensional cultures, which fail to replicate biological interactions among cells and between cells and the extracellular matrix (ECM), which occur in native tissues [31]. Conversely, tridimensional (3D) cell culture systems can mimic *in vivo* conditions involving cell-cell and cell-matrix interactions, such as dynamic regulation of signaling pathways and paracrine signals. Some examples of 3D culture systems include spheroids, tissue engineering constructs, and organoids [32].

Organoids are arranged structures, typically originated from stem cells, composed of multiple cell types that self-organize in culture, partly recreating tissue native architecture, morphology, and several biological interactions occurring *in vivo* [33, 34]. Although this research field has developed a lot in the last decade, especially after the iPSC development, organoid research dates back to the beginning of the 20th century. In 1910, Wilson demonstrated that disassociated adult cells contain enough information to reaggregate and self-reorganize into a specific multicellular structure resembling the original organ, without extracellular influence [35].

Organoid formation depends on the recapitulation of self-patterning, morphogenetic, and architectural rearrangements through manipulation of physical properties of the culture environment; endogenous and exogenous signals; and starting cell type culture with appropriate conditions [36]. During human embryonic development, there is a highly and tightly orchestrated differentiation process from zygote to self-organization of cells. In order to reproduce this process *in vitro*, iPSCs are induced to differentiate in specific lineages to form tissue-specific organoids with 3D biochemical cues [31].

Several parameters are controlled to stimulate self-renewal, differentiation, and self-organization [31]. The chosen organoid derivation method depends mainly on organoid type, on the required tissue differentiation, and on what is the ultimate practical application.

Organoids can be produced by self-assembly, when suspended cells self-organize in culture by cell aggregation through endogenous signals. Other strategies include starting induction with exogenous signals and then allowing self-organization of cells or providing exogenous factors continuously [36]. Differentiated stem cells can be seeded along with other cell types, such as endothelial and mesenchymal cells that, in combination, may form a 3D structure. In 2015, Takebe et al. published a generalized method for organ bud production from different types of tissues, in which mesenchymal stem cells (MSCs) were included into constructs. MSC-driven contraction was essential for organoids self-condensation, which could be reproduced for many cell types, such as liver, lung, heart, brain, and intestine cells [37]. In fact, the mesenchymal niche seems important for organoid engraftment and maturation after transplantation [38].

One important component of the organoid system is the ECM, which must support cell proliferation and enable cell adherence, diffusion of nutrients, and growth factors [39]. Stem cells must be in strict contact with ECM components, such as laminin, collagen, and fibronectin, important regulators of stem cell behavior, migration, and differentiation, especially through interaction with integrin receptors [40]. Matrigel, derived from murine cancer cell secretome [41], is widely used as a source of ECM for organoid manufacturing. However, there is a lot to lot variation, which brings an additional difficulty in standardizing culture conditions, and it may also trigger immunologic reactions. Some alternatives to delivery vehicles for organoid transplantation are being proposed, such as four-arm poly(ethylene glycol) (PEG) [42, 43] and Poloxamer 407, a triblock copolymer consisting of a central hydrophobic block of polypropylene glycol flanked by two hydrophilic blocks of PEG [44]. Single-cell genomics and clonal genome editing have made it possible to better understand cell behavior, cell-cell interactions, cell migration, and tissue organization, contributing to the generation of new ECM components compatible with organoid systems [45].

The immediate application for organoid technology is disease modeling and drug screening. The ultimate goal, given the promising application of organoids in regenerative medicine, is to perform transplantation of tissue-specific organoids to recover or improve tissue function. In this regard, some initial studies have been evaluating organoid transplantability. Transplants are being tested in mouse models, in which tissue engraftment, biocompatibility, and functionality are evaluated. Here, we review the main published works in this area, highlighting the main outcomes of intestinal, retinal, kidney, liver, pancreas, lung, brain, and heart organoid transplantation (Tables 1 and 2).

## 4. Transplantation of Organoids

### 4.1. Intestinal Organoids

In 2009, Clever and colleagues employed for the first time the concept of organoids when they noticed the proliferation and self-organization capacity of adult intestinal stem cells *in vitro* to form genomically stable 3D structures [73]. Ever since, there has been an increased investment in intestinal organoid production and optimization of culture condition differentiation and self-organization and many efforts to enable its transplantability, as numerous diseases, such as short bowel syndrome, Crohn's disease, and genetic intestinal diseases, can be treated by intestinal transplantation. However, there are still considerable issues, such as graft rejection, surgical complications, and risk of infection [74], revealing the need to create new strategies for intestinal organ replacement.

Many studies have attempted to evaluate the transplantability of intestinal organoids derived from adult or fetal mouse/rat intestinal cells [46, 47] or differentiated cells from PSCs [38, 49–51]. Intestinal epithelial organoids derived from mouse or rat adult intestine were orthotopically transplanted and showed successful engraftment and presence of enterocytes, enteroendocrine cells, Paneth cells, and goblet cells and reepithelization of damaged ileal mucosa [46]. Organoids derived from enhanced green fluorescent protein (EGFP^+^) mice, which were administered to immunocompromised mice with induced acute colitis, proved to be successful as it formed invaginated linings, cystic structures, and interacted with the mouse epithelium. Also, EGFP^+^ organoid transplantation regenerated colonic injured epithelium, improved body weight, and was capable of recovering the epithelial barrier function [47]. In this same study, it was demonstrated that EGFP^+^ mouse crypt cell organoids, derived from a single leucine-rich repeat-containing G-protein coupled receptor 5 (Lgr5^+^) stem cell, could engraft into mouse colon and remain with proliferative and cell differentiation capacity [47].

One of the first works reporting a functional human intestinal organoid transplantation using PSCs was done by Watson and collaborators in 2014. An intestinal organoid transplanted under the kidney capsule showed great engraftment and maturation, increasing in size and volume, and considerable vascularization. In addition, they reported an increased villus height, smooth muscle layer thickness, and crypt fission and depth, due to the release of humoral factors after ileocecal resection [38], hence proving that intestinal organoids respond to humoral factors released by the host and epithelium was capable of peptide uptake and presented an intestinal barrier. In 2015, using the same methodology for organoid production as Watson, Finkbeiner et al. performed a transcriptome-wide unbiased analysis of intestinal organoids, demonstrating successful engraftment *in vivo* and high expression of maturation markers (presence of Paneth cells and expression of *OLFM4*). Also, organoids acquired intestine architecture with villi containing lamina propria and had mesenchymal cells similar to adults [48].

In 2017, using an alternative source of ECM, Cruz-Acuña and collaborators developed an intestinal organoid with four-arm PEG macromer, with maleimide groups at each terminus, which, after 12 weeks, showed organoid growth (10- to 40-fold larger than the initial organoids), crypt-villus architecture, and regeneration of colonic wound, similar to results observed when these organoids were cultivated with Matrigel™ [49]. Moreover, to track the fate of intestinal organoids after transplantation, engraftment was evaluated by promoter-reporter biosensor in the lumen of mouse small intestine, using KLF5^mCherry^ or ISX^eGFP^ reporters that allow the monitoring of cell fate and differentiation *in vivo*. Results revealed fluorescent signals after three hours and as long as one week after transplantation, indicating successful organoid engraftment [50].

Most intestinal transplantation studies were performed using the kidney capsule as the transplantation site. However, mesentery transplantation of intestinal organoids represented a more physiologic strategy as it was observed 85% of engraftment into the host [51]. Also, a comparison between transplanted organoids after ten weeks and their *in vitro* counterpart revealed that organoid size and volume, as well as elements from epithelium, mesenchyme, and muscular layers, were larger. Histologically, organoids resemble human intestinal tissue, with specific cell lineages, subepithelial elements, and muscle, expressed intestinal maturation markers, and received vascular ingrowth from mesenteric vessels. This study was an important advance in this area as it created a model that may facilitate translational studies of intestinal organoid transplants [51].

Despite all the advances in the development of intestinal organoids, studies have mentioned that there are still limitations to overcome regarding intestinal organoid transplantation, such as (1) variation between intestinal organoid transplantation results from different rodents or species; (2) necessity to improve engraftment, intestine debridement, and organoid optimization; (3) difficulties to directly compare two models of transplantation (orthotopic versus ectopic); and (4) problems with functional significance of gene expression comparisons between distinct developmental stages.

### 4.2. Retinal Organoids

Retinal disorders (RD) are the main cause of vision loss and impairment, which are caused by loss/damage of photoreceptors. Over the years, many RD-related studies have been performed, especially with retinitis pigmentosa (RP) and age-related macular degeneration (AMD) [75]. Since there is no cure and accessible treatments for this type of disorder, there has been a great interest in developing methods for the transplantation of photoreceptor precursors or retina derivatives.

Many works were performed involving the transplantation of pluripotent cell derivatives (iPSC-derived retinal cells or human embryonic stem cell retina (hESC-retina)), most of which with promising and feasible results [76–83]. In this context, 3D cell culture systems have emerged as a model enabling the development of retinal tissue, grafts, and its derivative cells in substantial quantities for clinical transplantation tests [82–84].

The first protocol of retinal organoid was derived from mouse ESC by Eiraku and collaborators in 2011 [85, 86]. Later on, in 2012, Nakano and colleagues developed an ESC-derived retinal organoid, in which they not only reported that hESC-derived optic cup was larger than the one derived from mouse ESC (mESC) but also reported that hESC-derived neural retina grows into multilayer tissue containing rods and cones, while cone differentiation is rare in mESC culture [87].

Later on, with the advent of iPSC and 3D culture systems, the production of diverse retinal 3D structures from both mouse and human pluripotent cells was significantly improved [75, 84, 88–90]. In 2013, Gonzalez et al. performed transplantation of retinal organoids differentiated from embryoid bodies (EB) in *Gnat1^−/−^* mice (which exhibits stationary night blindness). In 2014, Assawachananont et al. performed the first transplantation of 3D retina sheets, derived from mESC and mouse iPSC, in *rd1* mice (a model with rapid and progressive RP). In the same year, Decembrini et al. developed a mESC 3D culture system to produce large amounts of photoreceptors. Once transplanted, 3D retina structures demonstrated maturation, morphological integration, production of new photoreceptors, integration with the outer nuclear layer (ONL) and outer segments, expression of phototransduction pathway proteins, and formation of synaptic connections [84, 89–91].

In 2016, Santos-Ferreira et al. developed mESC-derived retinal organoids, which were transplanted in the subretinal space of mice with either mild or severe cone-rod degeneration: *Prom1^−/−^* (prominin1-deficient) and *tg*(*Cpfl1;Rho^−/−^*) mice (a model generated from the crossing of cone photoreceptor function loss one mouse—*Cpfl1*—with rhodopsin knockout mice—*Rho^−/−^*), respectively. Organoids were capable of producing rod photoreceptors that, when transplanted in *Prom1^−/−^* mice, were able to integrate with the host's ONL, to maturate, survive, and express important proteins of the phototransduction pathway, as well as synaptic markers. On the other hand, in *tg*(*Cpfl1;Rho^−/−^*) mice, transplanted photoreceptors expressed rod markers but not synaptic markers and did not reach morphological maturation [56]. In 2017, Kruczek et al. produced organoids to obtain cone receptors, which are responsible for mediating high acuity and color vision during daylight. These mESC-derived organoids produced cone receptors that were transplanted into the subretinal space of *Aipl1^−/−^* mice (a model of end-stage retinal degeneration). Cone photoreceptors generated *in vitro* not only matured and survived within host eyes of both healthy and *Aipl1^−/−^* mice but also apparently made physical contact with inner retinal neurons. They also expressed synaptic transmission markers, as well as phototransduction-related proteins [57]. In 2018, McLelland et al. generated hESC-derived retinal organoid sheets, which were then placed within the subretinal space of *SD-Foxn1 Tg(S334ter)3Lav* (a model of severe RD immunodeficient nude rat). These transplanted retina organoid sheets exhibited maturation, integration, differentiation, production of functional photoreceptors and other retinal cells, synaptic activation, extensive transplant projections within the host RD retina, and improvement of PSC visual acuity and light sensitivity [58].

Even though these preclinical studies presented promising and extremely valuable results, they also pointed out limitations: (1) retinal organoids are composed of heterogeneous cell populations, which may represent a risk for tumor formation, cell contamination, and acute immune responses [56]; (2) the need for further investigation regarding the physiological functions of retina organoid-derived photoreceptors [57]; and (3) the absence of transplantation studies involving retina organoids derived from human iPSCs [75].

### 4.3. Kidney Organoids

A large number of patients with end-stage kidney disorders are dependent on hemodialysis and kidney transplantation [92]. Therefore, it is extremely relevant to invest in the production of transplantable kidney organoids. The kidney is a very complex organ, composed of many different cell types that, in order to perform its adequate function, need a complex 3D structure; thus, the development of organoids represents a valid investment [93].

One of the first attempts to transplant a kidney organoid dates back to 2012, when Xinaris and colleagues produced renal organoids derived from single-cell suspensions of E11.5 mouse kidneys and implanted them beneath the renal capsule of male athymic nude rats. These implanted kidney organoids exhibited formation of vascularized glomeruli with fully differentiated capillary walls, maturation of erythropoietin-producing cells, and physiological functions, including glomerular filtering and tubular reabsorption functions [59].

In 2014, Taguchi derived metanephric mesenchyme (MM) from mouse PSCs, which is responsible for generating many kidney components. This MM formed *in vitro* kidney 3D structures, such as vascularized nephric glomeruli and tubules [94]. Still in 2014, Takasato et al. differentiated hESCs into an *in vitro* self-organized nephron structure through simultaneous induction of MM- and ureteric bud-like (UB) progenitors [95]. In 2015, Morizane et al. developed multipotent hPSC-derived nephron progenitor cell differentiation, which were able to form nephron-like structures in both 2D and 3D culture systems. These organoids expressed podocytes, proximal tubules, Henle's loop, and distal tubule markers, resembling *in vivo* nephrons [96]. Next, in 2015, Takasato et al. generated kidney organoids containing nephrons with collecting duct network, early loops of Henle, and podocyte glomeruli [97]. In 2017, Taguchi et al. generated a kidney organoid derived from mPSC and hPSC by induction of MM and UB. This method enabled the development of a high-order architecture kidney organoid, which included peripheral progenitor niche and internally differentiated and interconnected nephrons [98].

Studies involving kidney organoid transplantation have started only recently. In 2018, hPSC-derived kidney organoid was transplanted under the renal capsule of immunodeficient mice. The transplanted kidney organoids exhibited maturation of podocytes, glomeruli vascularization, functional glomerular perfusion, and connection with preexisting vascular networks. Organoids, in the absence of any exogenous vascular endothelial growth factor, developed host-derived vascularization [60]. In 2019, Garreta et al. transplanted hPSC-derived kidney organoids into the chorioallantoic membrane (CAM) of chick embryos. CAM demonstrated to be a good microenvironment to study vascularization since it is not only a highly vascularized naturally immunodeficient soft environment but also easily manipulated and monitored. Besides, in parallel, hydrogel was also used, and they observed that kidney organoids transplanted into these soft environments stimulated organoids' differentiation and growth. CAM-transplanted organoids exhibited successful engraftment, vascularization, multiple blood vessels, and blood circulation [61]. Also, in 2019, Murakami et al. transplanted kidney organoids derived from mouse embryonic kidneys, under the renal capsules of immunodeficient mice. Transplantation results showed *in vitro* vascular development together with extensive UB branching and glomerulus formation, as well as formation and reestablishment of arteriolar network [62].

Although kidney organoid transplantation studies are still scarce, some challenges have already been pointed out and should be taken in consideration for future translational studies, such as (1) organoid size, as kidney organoids produced with larger amounts of cells presented higher survival rates [59]; (2) necessity to examine physiological functions (vascularization flow and urine production) in reconstituted kidneys [62]; and (3) the fact that the kidney is a highly complex and metabolic organ, therefore bioenergetics analysis should be considered with transplantation of kidney organoids [61].

### 4.4. Liver Organoids

The first functional liver organoid derived from pluripotent cells was made by Takebe et al. in 2015 [37]. The researchers used a coculture of hiPSC, human umbilical vein endothelial cells (HUVECs), and MSCs, which enabled the recapitulation of cell interactions during organogenesis, allowing them to self-organize into a 3D structure, resembling liver buds (iPS-LB) at the embryonic stage. When transplanted into nude mice, these liver buds exhibited quick and functional vascularization of the construct after 48 h of transplantation, evidenced by dextran infusion, showing functional human vessel formation and connections among donor and host cells. They also evaluated the number of vessels, which had already increased three days after transplantation, and the area of vessels, which was similar to the human liver. In addition, they evaluated drug metabolism activity, and the results were positive for this essential hepatic function and have rescued the drug-induced lethal liver failure model.

Despite their promising results, Song et al. (2015) argued that, for clinically relevant purposes, there was a need for researchers to use immunocompetent mice. Therefore, they decided to generate liver organoids with a slightly different protocol, combining initial 2D culture, to ensure homogenous distribution of nutrients and differentiation factors, with 3D culture, which allows complex interactions between cell-cell and cell-matrix to induce maturation. In order to transplant organoids into immunocompetent animals, they encapsulated the aggregates into biocompatible materials, such as alginate capsules. These capsules prevented direct immune cell rejection but did not eliminate immune response, as evidenced by detection of *Il-2*. Nevertheless, it did not compromise organoid function, maturation, and survival, as seen by the presence of albumin secretion and mature hepatic marker expression. However, one concern is fibrosis, which indeed occurred in a fraction of implanted capsules [44].

In 2018, Nie et al. investigated whether organoids could be used to treat acute liver failure in mice [55]. Considering future clinical applications, the group developed the liver organoid using three cell types originated from the same donor, unlike other published works that used different donors with different human leukocyte antigen types. After transplantation, organoids were able to perform hepatic functions and promote recovery from acute liver failure. Although very promising, further efforts are necessary to evaluate the use of single-donor cell-derived liver organoid for clinical treatment.

### 4.5. Pancreas Organoids

The development of pancreas organoids could represent a possible treatment for type I diabetes mellitus, an autoimmune disease in which destruction of pancreatic *β* cells results in insulin deficiency. However, most of the studies focus on cell therapy using only *β* cells. The generation of acinar and ductal cells from pluripotent cells, although poorly understood, has been successfully achieved through production of pancreatic organoids (PO) that were capable of expressing pancreatic markers and were functionally and ultrastructurally similar to the pancreas [52]. Orthotopic transplantation of these organoids exhibited engraftment after five weeks, neovascularization in the grafts, and expression of ductal and acinar markers and also validated the use of pancreas organoids to model cystic fibrosis.

Recently, Soltanian et al. proposed a strategy using PO to enhance maturation of pancreatic progenitors (PP) [53]. The PO was placed in a 3D-printed tissue trapper and heterotopically implanted into the peritoneal cavity of immunodeficient mice, and the results indicated that, in contrast to corresponding early PP transplants, 3D PO developed more vascularization as indicated by greater area and number of vessels, containing higher number of insulin-positive cells and displaying improved human C-peptide secretions. In another study, Lebreton et al. demonstrated that combining dissociated islet cells (ICs) with human amniotic epithelial cells (hAECs) into an organoid improves its vascularization, engraftment, and function *in vivo* [54].

### 4.6. Lung Organoids

Transplantation of lung organoids is a promising tool for airway diseases, such as asthma. These organoids can be formed by a 3D assembly of lung epithelial progenitor cells with or without mesenchymal cells [99], as well as by using adult stem cells and PSCs [70].

The first attempt to transplant lung organoids from human PSCs was performed by Dye et al. (2016), in which different conditions for transplantation were tested. Most of the transplants showed huMITO^+^ NKX2.1^+^ immature airway-like structures. The most successful transplants, in terms of organoid maturation, were lung organoids cultivated for one day in microporous polylactide-co-glycolide (PLG) scaffolds, which were able to engraft *in vivo*, differentiate into a similar airway epithelium, and generate secretory lineages, resembling the adult human lung [100].

The combination of adult bronchial epithelial cells, lung endothelial cells, and lung fibroblasts creates a human airway organoid suitable for ectopic transplantation: one week after lung organoid transplantation into the kidney capsule, Tan et al. (2017) observed proliferation of host cells in organoids' border and presence of human endothelial cells. Organoids reduced in size after six weeks; the vascular network was mainly of host origin, and *in vivo* environment stimulated maturation and switched to a nonproliferating status [70]. Similarly, Chen et al. were able to generate organoids with branching morphogenesis and proximodistal specification [69]. After 1.5 months of ectopic transplantation, lung organoids showed growth, tubular structure, and an airway epithelium formation. Branching structures and epithelial cells were observed after 5 months, and histology revealed multiciliated cells and similar morphology to proximodistal specification in lung branching.

In 2019, Weiner et al. developed an alveolar type 2 (AT2) organoid, which was then transplanted to influenza-infected mice. Thirteen days after transplantation, analysis revealed that AT2 organoids presented good engraftment *in vivo* and retained the AT2 fate. However, these organoids did not elevate the capability of oxygen exchange in the infected receiver mice and sometimes they adopt a dysplastic fate upon engraftment [71]. Dye and collaborators (2020) studied the efficiency and physicochemical properties of lung organoids generated in three different scaffolds: PLG scaffolds, PEG hydrogel, and polycaprolactone scaffolds. Although some scaffolds present some advantages compared to others, for instance, organoids developed in PEG scaffolds did not support maturation over eight weeks and increased immune cell recruitment, overall, lung organoid maturation is supported by multiple microporous scaffolds. The conclusion was that manipulation of scaffolds' physicochemical properties influences the explant's properties, directing tissue formation, and may be used for modeling normal development or disease states [72].

Some challenges of lung organoids transplantation are related to poor cell maturation, branching morphogenesis which appears to be random, and the mesenchyme nature and patterns that are not well understood [69].

### 4.7. Brain Organoids

One of the most difficult systems to understand is the cerebral, as it is a highly complex organ with many functionalities. Also, regular cell culture systems do not capture the organ's complexity and the access to material is difficult [101]. Therefore, the production of brain organoids is a promising tool to study and treat cerebral diseases, such as neurological diseases and mental disorders [102, 103].

In 2013, Lancaster and collaborators (2013) were able to derive brain tissue *in vitro* through a 3D culture system to study microcephaly. Previously, studies were performed with only neural tissue *in vitro*, and differently from other organs, there were no studies using whole-brain organoids until then [102].

After this study, many others were developed in order to enable the transplantation of brain organoids. In 2018, Mansour et al. generated the GFP hESC line from lentivirus-transduced human ESCs, which originated brain organoids after 40–50 days of culture. Only organoids that passed the quality criteria were implanted into a cavity in the retrosplenial cortex of nonobese diabetic-severe combined immunodeficient (NOD-SCID) mice [63, 102, 104]. Eight months after transplantation, cell differentiation and progressive maturation were observed, as well as synaptic connectivity between human axons and the host brain and axonal outgrowth in cerebral organoids. Researchers were also able to prove the organoid's successful vascularization through a cranial glass window that allowed tracing blood vessels. With these results, they were able to directly analyze the impact of environment and vascularization towards the brain organoids and verify their *in vivo* viability. The conclusion is that human brain organoids successfully interact with the mouse brain and present integration, maturation, and neuronal differentiation, which are promising for future human brain disorder treatment [63].

In 2018, Daviaud et al. compared cerebral organoids with neuronal progenitor cells (NPC), both derived from hESC. These organoids and NPCs were transplanted into the frontoparietal cortex of postnatal day P8-P10 mice. After two and four weeks of transplantation, they showed that brain organoids presented better results than NPCs, when comparing vascularization, graft survival, neural differentiation, and cytoarchitecture [64].

In 2019, Wang et al. developed and used cerebral organoids in the attempts of reversing damage after stroke. Parameters evaluated included the cerebral organoid volume, function recovery, effectiveness, and viability. Organoids were transplanted at 55 days in the rat middle cerebral artery occlusion, and results, 6 h–24 h later, demonstrated that cerebral organoids were able to differentiate and migrate into different brain regions. Also, they observed reduced brain damage volume, synaptic reconstruction, and neurological motor function recovery, among other neurological improvements, likely due to cell survival and vascularization, cell multilineage differentiation, and cellular replacement after stroke [65].

Recently, Dong et al. developed a protocol for the generation of small human brain organoids. After transplantation into the mouse medial prefrontal cortex, the authors observed that organoids survived and matured, extending 4.5 mm in length during the first engraftment. Differentiation of human cells into cortical neurons *in vivo* and electrophysiological activity affecting behavior were observed a few months posttransplantation. Organoid graft and host mouse brain interaction was also observed, involving synaptic connections and a possible functional integration between them [66].

Even though many improvements towards transplantation of cerebral organoids have been made, there are still some concerns, such as (1) the ethical implications related to the creation of brain chimeras that, somehow, could be responsible for “humanization” of host animals, raising questions about brain development and function [105]; (2) limited formation of neuronal circuits, microenvironment, immune system, and vascular circulation, as the absence of oxygen can interfere in the neuronal development and migration [63]; and (3) difficulty of tissue cross-communication and organization of the brain shape and structure [31].

### 4.8. Heart Organoids

Cardiac organoid production is still an area poorly explored. One advantage of 3D cultures for cardiac disease treatment is the possibility of observing tissue dynamics and organ physiology.

In 2019, Varzideh et al. developed the first hiPSC-derived cardiac organoid for transplantation. After 24 h of organoid formation, the presence of three different cell types was observed, cardiac progenitor cells (CPC), MSCs, and endothelial cells. These cells started to self-organize into 3D organoids after 72 h, and after one week, cardiac organoids presented a homogeneous beating, which maintained organoids mechanically stable for transplantation [67]. Detection of cardiomyocyte (CM) maturation markers and electrophysiological activity study were also evaluated before transplantation. To assist *in vivo* transplantation, a two-piece basket was fabricated using a 3D printer, and collagen type I was used to encompass the cardiac organoids, which were then transferred into the basket [67]. The transplantation was performed on the internal abdominal muscle of male nude mice, and four weeks later, organoids revealed extensive neovascularization, highly organized sarcomeric structures, CM marker expression, and electrophysiological activity. This *in vivo* transplantation induced structural organization of myofibrils, enhanced gene expression, and excitation-contraction coupling. CPCs interacting with mesenchymal cells developed into CMs and other specialized cells, allowing primary heart organogenesis. To facilitate organogenesis and because of their immunomodulatory and anti-inflammatory properties, MSCs were also included [67]. COs from transplanted mice were detached from the basket and transferred to a chick embryo to complete the lymphoid system development [67].

In conclusion, complex organoids are a promising tool to model heart diseases for regenerative medicine and drug testing, but further challenges still need to be overcome, due to (1) heart system complexity and diversity; (2) functional human cardiac organoids requiring at least three cell types: cardiomyocytes, cardiac fibroblasts, and cardiac endothelial cells [106]; and (3) improvement of cell maturation, as iPSC-derived cardiomyocytes, even after *in vitro* differentiation, still have embryonic properties [107].

## 5. Challenges on Organoid Transplantation

Current strategies for treatment of organ failure diseases involve transplantation of existing organs, cell therapy, and regenerative medicine concepts. The organoid system has arrived as an important alternative that is capable of recapitulating embryonic development, creating a favorable microenvironment to derive complex and functional structures resembling an organ. Here, we have reviewed the first attempts to generate different organoid systems, using animal models to evaluate their transplantability.

In general, preclinical evidence supports positive engraftment of organoids after transplantation, once it has been observed that these 3D structures integrated, maturated, vascularized, and developed specific targeted tissue physiological functions. Nonetheless, there are important subjects that must be taken into account before their application in organ failure diseases [45].

### 5.1. Organoid Size

One crucial issue regarding organoids for transplantation purposes is their small size. Thus far, organoids measure typically 10 *μ*m to 1 mm in diameter, but there have been some attempts to make them bigger. One approach to solve this issue is using spinning bioreactors, thereby facilitating oxygen and nutrient absorption, to make larger brain organoids resembling more of a human organ, for instance [102, 108]. Another option is combining small organoids to make a larger one as it was made for epithelia-only gut organoid [109].

The size is a major concern in some specific organs, such as kidney organoids, in which bigger organoids, produced with more precursor cells, had more chances of survival and growth than the smaller ones [59]. In contrast, the large size of organoids may be a problem. Human cerebral organoids seem fragmented after two weeks, maybe because of disparity in the size of organoid and host brain or due to hypoxia [64].

### 5.2. Cell Maturation

Cell maturation is important to ensure organoids will execute tissue-specific functions and guarantee their safety and efficiency after *in vivo* engraftment. For example, in some cases, differentiation protocols yield cells more similar to fetal than to adult ones, which might not be suitable for tissue replacement intents [44].

On the other hand, it seems that organ buds formed by less mature tissues might be a better strategy toward regeneration after transplantation, which is shown by some of the reviewed works: with kidney organ bud experiments [37], with intestinal organoid [38], and with heart organoid [67]. It occurs because the *in vivo* environment provides biochemical and physical signals from multiple sources, as well as vascularization and innervation networks that are difficult to completely reproduce *in vitro*. Besides, transcriptome-wide comparisons between intestinal organoids cultivated only *in vitro* or transplanted to NSG mice showed that *in vivo* engraftment improved cellular differentiation and organoids resemble mature adult-like intestine tissue, while *in vitro* organoids were more similar to fetal tissue [48].

Another aspect that influences cell maturation is the microenvironment in which the organoids are cultivated. For instance, Garreta et al. demonstrated that kidney organoids in soft environments, such as hydrogels or CAM, enhanced its formation and growth [61]. Also, in Völkner et al.'s study, the authors mentioned that several processes, such as progenitor proliferation and cell differentiation, are potential sources for organoid variation [110].

### 5.3. Animal Models

Several preclinical trials are required to confirm the true potential of organoids as a medical device to replace or improve organ function. However, these *in vivo* tests involve many concerns and difficulties in translation for human application. For example, according to Avansino et al., there is considerable variation between distinct rodent models and species, which makes it difficult to establish an ideal animal model [46]. Translational studies are needed to achieve successful clinical application, and it is important to count with larger animal models to better reproduce human conditions [51].

In addition, most of these studies still rely on immunodeficient models, because in general, organoids are derived from human cells, and this could introduce an important experimental variation, since mice's immune system would most likely reject the transplanted organoid. Only one out of the articles reviewed here used an immunocompetent animal and encapsulated the organoids in alginate which partially avoided immune system cell attack [44]. Nevertheless, this approach is still a xenotransplant and cannot simulate the clinical scenario of allogeneic transplantations.

One strategy to overcome this limitation is the use of humanized animal models, which have already been developed elsewhere [111]. Also, it is important to use larger animal models, such as pigs, to better understand possible outcomes of organoid transplantation [45].

### 5.4. Site of Transplantation

The site of transplantation must be chosen carefully. Fetal intestine organoids did not survive transplantation under the kidney capsule, showing that orthotopic transplantation could be more suitable [100]. Also, lung organoids did not survive after transplantation into the kidney capsule [68].

On the other hand, the kidney capsule is often chosen, because it is an isolated location, with a certain degree of immune privilege, good accessibility, and transplantation which is usually well tolerated by the host [51]. However, as discussed by Cortez and collaborators, some limitations regarding the kidney capsule for intestinal organoid transplantation made them search for closely related sites for intestinal transplantation, in this case, the mesentery [51].

In two works related to kidney and heart organoids, the site of transplantation differed from the usual, kidney capsule [61, 67]. They used chick CAM, which demonstrated to be highly vascularized, as well as a naturally immunodeficient and easier to monitor microenvironment [61].

Further alterations were done to facilitate organoid transplantation and recovery. For example, in heart organoids, they used a 3D printed basket [64], and for pancreas organoids, tissue trapper was used [53].

### 5.5. Vascularization and Innervation

Organoid vascularization is a critical issue because the absence of vascular networks limits organoid growth and factor exchange, reducing nutrient distribution [31, 93]. Using endothelial cells as an organoid component is a suitable strategy. HUVECs present in the liver bud organoids were capable of engrafting and forming blood vessels [112]. However, most *de novo* vascularization that occurs into organoids after transplantation is derived from host cells.

One option to investigate vascularization was transplanting organoids into CAM. Both studies that used CAM generated positive results, since immunofluorescence analysis and fluorescent isothiocyanate-dextran confirmed the presence of chick blood vessels and blood circulation [61, 67].

An important aspect that was not investigated by either of the works presented here is innervation, which is essential for the proper control of organ functions.

### 5.6. Follow-Up after Transplantation

An important matter for organoid transplantation technology is tracking organoids *in vivo* to evaluate their behavior, engraftment, vascularization, and function. Development of iPSC-expressing fluorescent biosensors through lentiviral vector infection, for example, enable the visualization and study of organoids inside the host, creating an efficient and informative tracking system using tissue-specific promoters [50].

Another crucial aspect of organoid transplantation safety is to make sure that no tumor is formed, since tumorigenicity is a clinical hurdle for PSC-based therapies [113]. In some cases, fibrosis formation was a concern, in particular in those protocols that used encapsulation of organoids using biocompatible materials [44].

Despite all of these challenges, organoid transplantation represents a growing promising system for regenerative medicine application. The first-in-human trial of intestinal organoids is being planned to be carried out by Tokyo Medical and Dental University (TMDU) for treatment against inflammatory bowel disease. Besides that, the INTENS team is leading a research with adult stem cells to treat short bowel syndrome (SBS). In the meantime, diagnostic tools have been developed by a group called Hubrecht Organoid Technology (HUB). The purpose of these tools is to link patient-specific genetic and phenotypic information. A center in Yokohama City University (YCU) was investing in a treatment of pediatric metabolic liver disease. Also, in Cincinnati Children's Hospital Medical Center, a Center for Stem Cell and Organoid Medicine (CuSTOM) was created, encompassing various collaborations focused on organoid research [45].

## 6. Final Remarks and Conclusions

Organoids are promising tools for disease modeling, drug screening, and personalized medicine. The ultimate application of organoid technology is to use them for organ regeneration and replacement therapies, reducing whole organ transplant requirements and improving the life quality of patients. The therapeutic use of organoids would be an alternative to the challenging transplantation of organs with a short period of viability outside the body, such as the heart and lungs. In particular, organoids should highly impact regenerative treatments of organs that remain technically nontransplantable, such as the brain. The recent development of edited pluripotent stem cells with targeted disruption of HLA genes by CRISPR/Cas technology should also facilitate the generation of immunocompatible healthy organoids for widespread therapeutic purposes.

Compared with typical cell cultures, organoids better reproduce the structural complexity of a real organ, recreating tissue native architecture, morphology, and several biological interactions occurring *in vivo*. Despite being still in its infancy, organoid transplantation for the intestine, retina, kidney, liver, brain, heart, pancreas, and lung seems feasible and safe, based on preclinical evidence showing engraftment and great biocompatibility. After transplantation, studies have shown that organoids generate differentiated and functional cells that are capable of interacting with other host cells. Taken together, the good outcomes of these initial studies encourage the exploration of organoids for regenerative medicine purposes. However, relative organoid graft immaturity compared with host natural organ, incomplete functional tissue integration, and possible occurrence of heterotypic cell interactions are some of the remaining challenges to overcome before clinical application.

## Figures and Tables

**Table 1:** Description of main studies performing organoid transplantation.

Organ	Ref.	Cell source	Receiver	Extracellular matrix	Time of evaluation after transplantation
Intestine	[46]	Rat/mouse neonatal small bowel	Adult male Lewis rat or wild-type mice	Extracellular matrix gel	2, 3, or 6 weeks
Intestine	[47]	EGFP^+^ mouse crypt cells	Immunocompromised Rag2^–/–^ mice	Matrigel-containing PBS	6 d, 16 d, 4 weeks
Intestine	[38]	Human ESCs or iPSCs	NSG IL2Rg-null mice	Type I collagen	6 weeks
Intestine	[48]	H9 human ESCs	NSG IL2Rg-null mice	Type I collagen	16 weeks
Intestine	[49]	Human ESCs or iPSCs	NSG IL2Rg-null mice	PEG-4MAL	12 weeks
Intestine	[50]	Human iPSCs	NSG IL2Rg-null mice	Matrigel	1 week, 3 h
Intestine	[51]	H1 ESC	NSG IL2Rg-null mice	Matrigel	10 weeks
Pancreas	[52]	hPSCs	Immune-deficient mice	Matrigel	5 weeks
Pancreas	[53]	hESC	Nude mice	Growth factor-reduced Matrigel	30 d, 60 d, 90 d
Pancreas	[54]	ICs and hAECs	Diabetic SCID mice	Agarose	1 month
Liver	[37]	hiPSC, HUVEC, MSC	NOD/SCID mice	Matrigel diluted with EGM	Multiple time points, ranging from 0 to 60 days
Liver	[44]	iPS-H and stromal cells	C57BL/6 mice	2D: Matrigel; 3D: Pluronic f127; and transplant: alginate to encapsulate	Twice a week, 3 d postoperation until day 24
Liver	[55]	hiPSC endoderm, EC and MSC	Alb-TRECK/SCID mice	Growth factor-reduced Matrigel diluted with SFD medium	Every 5 d until the 20th day
Retina	[56]	Wild-type E14TG2a mES	Prom1^−/−^ and tg(Cpfl1;Rho^−/−^) mice	Growth factor-reduced Matrigel	3 to 4 weeks
Retina	[57]	mESC (E16 CEE and Crx-GFP line)	Wild-type and Aipl1^−/−^ mice	Growth factor-reduced Matrigel	3 weeks
Retina	[58]	hESC	SD-Foxn1 Tg(S334ter)3Lav	Growth factor-reduced Matrigel	54 to 300 d
Kidney	[59]	Single-cell suspensions derived from E11.5 CD1 mouse kidneys	Male athymic nude rats	—	3 and 6 weeks
Kidney	[60]	hESC and hPSC	NOD/SCID mice	Vitronectin-coated culture dishes	7 d and 28 d
Kidney	[61]	hPSC	CAM of 7-day-old chick embryos	Vitronectin-coated culture dishes	3 to 5 d
Kidney	[62]	E11.5 mouse embryonic kidneys	NOD/SCID mice	Atelocollagen membranes	7 d
Brain	[63]	hPSC	NOD/SCID mice	Matrigel	0.5–8 months
Brain	[64]	hESC or hiPSC (H9 hES cells, WAe009-A)	P8-P10 CD1 mice	Matrigel	In 2 and 4 weeks
Brain	[65]	hESCs	Sprague-Dawley rats	Matrigel	4 weeks
Brain	[66]	hESCs and hiPSCs	SCID mice	—	1–5 months
Heart	[67]	hESC coculture with hESC-MSC, CPC, and EC	Male nude mice (25–30 g, B6NU)	Matrigel	12.5 d, 4 weeks
Lung	[68]	hESCs	NSG mice	With or without PLG and/or Matrigel	4, 6, 8, 12, or 15 weeks
Lung	[69]	hESCs and iPSCs	NSG mice	Matrigel	1.5, 5, or 7 months
Lung	[70]	HBEpC, HMVEC-L, and HLF	NSG mice	Matrigel	1 or 6 weeks
Lung	[71]	CD45^−^ EPCAM^+^*β*4^−^ AT2 cells	Influenza-infected mice	Matrigel	13 d
Lung	[72]	hESCs	NSG mice	PEG, PLG, and PCL	Between 1 and 8 weeks

Abbreviations: (h/m) ESC: human/mouse embryonic stem cells; Aipl1^−/−^ mice: a model of end-stage retinal degeneration; AT2: alveolar type 2 cells; CAM: chick chorioallantoic membrane; Crx-GFP ESC lines: ESC lines of transgenic mouse line expressing GFP with control of endogenous photoreceptor-specific promoter Crx; d: day(s); EC: endothelial cells; h: hour(s); hAECs: human amniotic epithelial cells; HBEpC: human bronchial epithelial cells; HLF: human lung fibroblasts; HLO: hPSC-derived lung organoids; HMVEC-L: human microvascular lung endothelial cells; ICs: islet cells; iPS-H: human induced pluripotent stem cell-derived hepatocyte-like cells; iPSCs: induced pluripotent stem cells; MSCs: mesenchymal stem cells; NSG mice: nonobese diabetic/severe combined immunodeficient (NOD/SCID) mice; PBS: phosphate-buffered saline; PCL: polycaprolactone; PEG: poly(ethylene glycol) hydrogel; PEG-4MAL: four-arm poly(ethylene glycol) (PEG) macromer with maleimide groups at each terminus; PLG: poly(lactide-co-glycolide) scaffolds; Prom1^−/−^ mice: prominin1-deficient mice; PSC: pluripotent stem cells; SC: superior colliculus; SD-Foxn1 Tg(S334ter)3Lav: severe retinal degeneration immunodeficient nude rat; tg(Cpfl1;Rho^−/−^) mice: cone photoreceptor function loss 1 (Cpfl1) crossed with rhodopsin knockout mice (Rho^−/−^).

**Table 2:** Description of main studies performing organoid transplantation.

Organ	Ref.	Methods to evaluate engraftment, maturation, organoid behavior, and physiologic responses	Site of transplantation (orthotopic or ectopic)	Limitations
Intestine	[46]	Bile acid uptake; HS; IHC; GFP^+^ mouse-derived organoids	Orthotopic—omentum	Variation between different rodents or species; improvement of engraftment and intestine debridement needed
Intestine	[47]	MV; TRITC-dextran analysis; EGFP^+^ cells; IM; body weight	Orthotopic—colon	Optimization needed
Intestine	[38]	MV; HS; IM; qPCR; TEM; LGR5 reporter; permeability; peptide uptake	Ectopic—kidney capsule	—
Intestine	[48]	qPCR; HS; TEM; IM; RNAseq	Ectopic—kidney capsule	It is unclear if gene expression variation between distinct development stages has truly functional significance
Intestine	[49]	MV; FM of mCherry expressing organoids; HS; IM; wound closure quantification; *in situ* hybridization	Ectopic—kidney capsule	—
Intestine	[50]	iPSC lines expressing reporters for *ex vivo* FI; HS; live-cell imaging	Ectopic—kidney capsule/orthotopic—intestinal lumen	—
Intestine	[51]	Survival rate; percent of engraftment and size of organoids; IHC; HS	Orthotopic—mesentery	Impossibility to directly compare two models of transplantation; level of organoid functionalization and maturation was not evaluated
Pancreas	[52]	MV; IF for human origin marker; trilineage differentiation potential; HS; IF for acinar and ductal markers	Orthotopic	
Pancreas	[53]	Insulin IHC; human C-peptide serum measurement in PO, ES-PP, and ECM; vessel area of harvested grafts and vessel numbers	Ectopic—intraperitoneal cavity	—
Pancreas	[54]	Blood glucose measurements; IM; qPCR; IHC; human C-peptide serum measurements	Ectopic—under the kidney capsule	Significant islet loss in the early posttransplant period
Liver	[37]	MV; dextran infusion at day 3; connections' visualization among HUVECs and host vessels; quantification of human vessels; functional vessel length between human iPSC-LB x HUVEC human MSC transplants	Ectopic	—
Liver	[44]	ELISA; qPCR; IM; IHC	Ectopic—intraperitoneal cavity	Cell encapsulation did not completely eliminate the immune responses induced by foreign cells; fibrosis was reported. Further work is needed to develop iPS-H for clinical uses
Liver	[55]	MV; ELISA; IHC; IF analysis; cytochrome P450 3A4 and urea assay	Ectopic—renal subcapsule space	Further efforts are necessary to evaluate the use of SDC-LOs in clinical treatment
Retina	[56]	IHC; IM assays; retinal sections; expression of phototransduction and synaptic markers; ERG measurements	Orthotopic—subretinal space	Photoreceptor replacement procedures need to be optimized; risk of initiating tumor growth; proper differentiation and sorting methods aimed at specific target cell types are needed, as well as long-term studies to assess safety, and development of strategies to promote synapse formation and potential functional repair
Retina	[57]	IM assays; FC; GFP measurement	Orthotopic—superior and inferior hemispheres of the eye (subretinal space)	Further investigation of potential functionality of the transplanted cells
Retina	[58]	OKT response testing and SC electrophysiological recording; IHC for donor and retinal markers; spectral-domain OCT imaging and quantification	Orthotopic—subretinal space	Improve retina transplant lamination
Kidney	[59]	IHC; IF; IM assays; molecules' expression to assess maturation; VEGF injection; CM	Orthotopic—beneath the renal capsule	Ethical concern regarding the use of exogenous spinal cord cell layer; draining collection system is needed, as well as further maturation techniques to obtain a more robust collecting system and excretory function
Kidney	[60]	IF; nanoelectron microscopy; *in vivo* imaging; IM; SEM analysis; repeated intravital multiphoton imaging; TEM	Orthotopic—under renal capsule	Development of a glomerular filtration unit is needed
Kidney	[61]	*In vivo* injection of dextran–FITC into the CAM; IF analysis; IHC; TEM analysis	Ectopic—CAM of chick embryos	Development of methods to improve organoid differentiation (*in vivo* or *in vitro*), such as biomimetic approaches, is needed
Kidney	[62]	Whole-mount and section staining; FC	Orthotopic—under renal capsules	Formal proof using dye injection into the host circulation and examination of physiological functions in reconstituted kidneys are needed; differences between transplanted organoids and branching patterns of intrarenal arterioles from *in vivo* kidneys
Brain	[63]	GFP^+^ detection; neuroepithelial ventricular zone analysis; level of gliogenesis; IM; axonal outgrowth and synaptic connectivity analysis; cranial glass window; two-photon calcium imaging; electrophysiological with cross-correlation; optogenetic control	Orthotopic—retrosplenial cortex	Improvements in vascular system, neuronal circuits, and immune system are needed, as well as understanding the complex physiological context of the brain
Brain	[64]	Fluorescent protein; ICC; GPF expression; cerebral organoid and the graft area measurements; blood vessels and microvasculature quantification; IHC; IM; neuronal differentiation	Orthotopic—frontoparietal cortex	Technical difficulties or increased cell death before engraftment; controlling stem cell proliferation after engraftment and developing a more complex cerebral organoid are needed; ethical concerns
Brain	[65]	IF; IHC; behavior tests (dysfunction, mNSS); image quantification; measurement of neural connectivity and brain functionality	Orthotopic—middle cerebral artery	—
Brain	[66]	HS; IM; FI; cell morphology; photostimulation of grafted cells	Orthotopic—medial prefrontal cortex	—
Heart	[67]	Beating; voltage-sensitive dye imaging; vasculogenesis; neovascularization; IM; organization of sarcomeric structures; RT-qPCR	Ectopic—internal abdominal muscle with a basket	Maturations details (pre- and posttransplant)
Lung	[68]	IM	Ectopic—kidney capsule, omentum, or fat pad	Additional cues for tissue maturation are needed, as well as variability across transplants
Lung	[69]	IF; HS; dot blot	Ectopic—kidney capsule	Terminal maturation; branching seems random; nature of mesenchyme is unclear; *in vitro* culture biases to restricted cell types
Lung	[70]	IM; size evaluation; proliferation	Ectopic—kidney capsule	Ectopic transplantation is limited and does not resemble true regenerative potential
Lung	[71]	IM; pulse oximetry; qPCR	Orthotopic	Better elucidation regarding transcriptional changes and signals in AT2 transplanted organoids; better optimization of organoid transplant
Lung	[72]	IHC; H&E; imaging	Ectopic—epididymal blood vessels and fat pad	PEG did not support maturation over the 8 weeks; increase in immune cell recruitment in PEG scaffolds due to hydrogel swelling

Abbreviations: CM: confocal microscopy; ERG measurements: electroretinogram; FC: flow cytometry; FI: fluorescence imaging; FITC: fluorescein isothiocyanate; FM: fluorescence microscopy; (E)GFP: (enhanced) green fluorescent protein; H&E: hematoxylin and eosin staining; HS: histology; ICC: immunocytochemistry; IF: immunofluorescence; IHC: immunohistochemistry; IM: immunostaining; LGR5: leucine-rich repeat-containing G-protein coupled receptor 5; MV: macroscopic view; OKT: optokinetic response; qPCR: quantitative polymerase chain reaction; RNAseq: RNA sequencing; SEM: scanning electron microscopy; TEM: transmission electron microscopy; TRITC: tetramethylrhodamine isothiocyanate.
